# Does plant community plasticity mediate microbial homeostasis?

**DOI:** 10.1002/ece3.6269

**Published:** 2020-04-24

**Authors:** Kate M. Buckeridge, Jennie R. McLaren

**Affiliations:** ^1^ Global Academy of Agriculture and Food Security The Royal (Dick) School of Veterinary Studies University of Edinburgh Edinburgh UK; ^2^ Department of Biological Sciences University of Texas at El Paso El Paso TX USA

**Keywords:** Arctic tundra, carbon use efficiency, extracellular enzymes, long‐term fertilization, plant–microbe interactions, stoichiometry

## Abstract

Microbial homeostasis—constant microbial element ratios along resource gradients—is a core ecological tenet, yet not all systems display homeostasis. We suggest investigations of homeostasis mechanisms must also consider plant–microbial interactions. Specifically, we hypothesized that ecosystems with strong plant community plasticity to changing resources will have homeostatic microbial communities, with less microbial resource cost, because plants reduce variance in resource stoichiometry. Using long‐term nutrient additions in two ecosystems with differing plant response, we fail to support our hypothesis because although homeostasis appears stronger in the system with stronger plant response, microbial mechanisms were also stronger. However, our conclusions were undermined by high heterogeneity in resources, which may be common in ecosystem‐level studies, and methodological assumptions may be exacerbated by shifting plant communities. We propose our study as a starting point for further ecosystem‐scale investigations, with higher replication to address microbial and soil variability, and improved insight into microbial assimilable resources.

## Graphical abstract

We explore if plant community response to changing resource supply influences microbial efforts to maintain homeostasis. Our conflicting results led to a new framework for considering microbial homeostasis and suggestions for future studies.

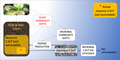

## 1 INTRODUCTION

Organismal homeostasis is defined as consistency in element composition despite fluctuations in environmental resource availability (Sterner & Elser, 2002). This is a core tenet of ecological stoichiometry, a conceptual framework that explains how element proportions drive processes in organisms and ecosystems. In plant–soil–microbial systems, microbial homeostasis determines rates of decomposition, nutrient retention and biomass production (Zechmeister‐Boltenstern et al., 2015), processes that predict food security, fertilizer pollution, and carbon storage (Paustian et al., 2016). Uncertainty in these predictions arise because microbial homeostasis can be both strong or weak, in other words, microbial C:N:P is sometimes invariant, and sometimes varies with resource C:N:P. Despite increasing attention to the mechanisms that support microbial homeostasis, it remains unclear what causes this variation.

The current paradigm for exploring microbial homeostasis was nicely outlined by Mooshammer, Wanek, Zechmeister‐Boltenstern, and Richter (2014), with a four‐option mechanistic framework describing how microbial communities can respond to the stoichiometric imbalance between their biomass and their resources at the individual and or community level. Microbes may: (1) store C, N or P or shift community structure to match their biomass composition toward their resource; (2) mobilize needed resources by enhancing extracellular enzyme activity (EEA); (3) alter element use efficiencies (the ratio of the investment of an element in growth versus. the total uptake of this element) by excreting nutrients in excess; or (4) alter resource pools via inputs of nutrients external to the measurement system, such as by N‐fixing prokaryotes or fungal hyphae. There was an earlier focus in modeling and experimental research on EEA as the dominant mechanism (Sinsabaugh, Hill, & Follstad Shah, 2009), while more recent studies suggest that changing nutrient use efficiency is the primary mechanism (Fanin, Moorhead, & Bertrand, 2016; Manzoni et al., 2017; Mooshammer et al., 2014). Here, we propose that mechanistic studies of microbial homeostasis at the ecosystem scale—with associated higher complexity of interactions—should also consider plant community mechanisms that alter the soil resource pool.

Changing plant community structure may influence nutrient outputs, inputs and the associated microbial community, three mechanisms that could change how the microbial community maintains, or fails to maintain homeostasis. *Nutrient outputs:* plant functional types take up nutrients in different ratios (McLaren & Turkington, 2010). *Nutrient inputs*: changing soil resources changes the stoichiometric ratio of individual plant tissue (Shaver & Chapin, 1980) and the community averaged plant tissue stoichiometry, through shifts in plant functional group composition (Guiz et al., 2016). Individual and community tissue stoichiometry alters litter and root exudate stoichiometry, and the litter:exudate ratio of these inputs, the latter which may further change microbial mechanisms of C and nutrient acquisition (Sokol et al., 2018). *Plant–microbe associations*: not all microbial populations maintain homeostasis (Danger, Gessner, & Bärlocher, 2016) so when plant community shifts are associated with microbial community shifts, this may lead to changes in the preferred mechanisms or ability to maintain homeostasis.

Soil type is the dominant control on microbial community structure and activity, with both plant community and resource supply secondary controls (Fierer, 2017). Studies that examine microbial efforts to maintain homeostasis under various resource regimes have been most commonly conducted with both soil type and plant communities varying (Cleveland & Liptzin, 2007; Fanin et al., 2016; Nottingham et al., 2015; Sinsabaugh et al., 2009; Tipping, Somerville, & Luster, 2016), or in mesocosms on the same soil type with the same (or no) plant community (Heuck, Weig, & Spohn, 2015; Joergensen & Scheu, 1999; Pinsonneault, Moore, & Roulet, 2016; Zhou, Wang, & Jin, 2017). In ecosystem‐scale investigations of homeostasis, both the microbial community and the plant community respond to the changes in resource availability, although not always in tandem, while soil type usually remains constant. We are not aware of examinations of microbial homeostasis using experimental nutrient additions resulting in changing plant functional groups on the same soil type, despite that this is a likely outcome of enhanced nutrient pollution in natural ecosystems (Dormann & Woodin, 2002; Suding et al., 2005; Xia & Wan, 2008).

We addressed this research gap by investigating microbial homeostasis and potential mechanisms—including the role of plants—for ecosystem response to stoichiometric imbalance in long‐term fertilization experiments in two dominant, close‐proximity (<2 km separation), upland Arctic tundra ecosystems: moist acidic tundra (MAT) and moist nonacidic tundra (MNT). Despite similar vegetation functional groups, vegetation community response to fertilization differs between the sites. The MAT has responded to fertilization with large increases in *Betula nana*, a deciduous shrub known for its plastic response to nitrogen and phosphorus additions (Bret‐Harte, Shaver, & Chapin, 2002). In contrast, the MNT initially responded to fertilization with increases in a variety of functional groups, particularly forbs and grasses (Hobbie, Gough, & Shaver, 2005), and in the longer term responded primarily with reductions in moss and few changes in vascular plants (McLaren & Buckeridge, 2019). We use these two sites to test the hypothesis that a large plant community response to fertilization can minimize microbial effort while still maintaining microbial homeostasis across a steep gradient of resource supply.

## 2 SITE DESCRIPTION AND METHODS

### 2.1 Study site

The study was conducted at the Arctic LTER site at Toolik Lake in northern Alaska, USA (68°38’N and 149°43’W, elevation 760 m) in the MAT and MNT (described previously in McLaren and Buckeridge 2019) which are dominant ecosystems of the Alaskan tundra. The two ecosystems differ based on age and acidity: The MAT site is on older substrate (50–120 k y) with a pH = 3–4, and the MNT is on younger substrate (11.5–25 k y), with a neutral pH.

### 2.2 Experimental design

We sampled existing long‐term fertilization experiments established and maintained by the Arctic LTER in both vegetation types. Fertilization treatments in both experiments represent a full‐factorial addition of *N* (10 g/m^2^/yr as NH_4_NO_3_) and P (5 g/m^2^/yr as P_2_O_5_), with fertilizer applied annually in pellet form following snowmelt (early June) for 26 years (MAT, established in 1988) and 16 years (MNT, established in 1997). From each experiment, we sampled a single 5 × 20 m plot from each of the three treatments (N, P, N + P) and the control, from each of four (MAT) or three (MNT) replicate blocks.

### 2.3 Vegetation and soil sampling

Aerial percent cover of mosses, lichens, litter, and all vascular plant species was visually estimated in each treatment plot in mid‐July 2013, within eight −1 m^2^ adjacent quadrats in each plot. Vole litter was assigned visually as the haying/nesting activities of small mammalian herbivores, primarily *Microtus oeconomus* and *M. miurus.* A ca. 10 × 10 cm column of soil was collected from each MAT and MNT plot to the depth of the permafrost in early July 2013. All organic horizons were <20 cm deep and were separated into the upper organic (0–5 cm depth) and lower organic (>5 cm depth) layers. Soils were homogenized and all large roots (>1 mm diameter) removed in the field laboratory. Soil was then partitioned for the analyses below, frozen at −20°C and shipped for analyses.

### 2.4 Soil and microbial biomass extraction and analysis

Field‐moist and thawed soil samples (10 g) were shaken with 40 ml of ultrapure water or with water plus CHCl_3_ (Fierer, Schimel, & Holden, 2003). Extractable organic C (EOC) and total *N* (ETN) and PO_4_–P contents in the CHCl_3_ and non‐CHCl_3_ extracts were determined as described previously (McLaren & Buckeridge, 2019).

### 2.5 Soil microbial extracellular enzyme analysis

We assayed for the activity of three hydrolytic enzymes that release C, N, and P at the terminal stages of organic matter decomposition: cellulose‐degrading beta‐glucosidase (BG), chitin‐degrading N‐acetyl‐glucosaminidase (NAG), and phosphatase (AP), using standard methods, as described previously (McLaren & Buckeridge, 2019).

### 2.6 Data analysis and statistical models

The two ecosystems have different lengths of time under experiment and were thus evaluated separately and the results qualitatively compared. For all soil analyses, data from both organic horizons (upper and lower) were pooled, with the values weighted by the depth of each horizon. Microbial biomass C, N, and P flushes (hereafter, MBC, MBN, and MBP) were calculated as the difference between EOC, ETN or PO_4_–P in CHCl_3_ and non‐CHCl_3_ extracts, with no correction factor for incomplete CHCl_3_‐release applied. For potential enzyme activity (EEA), for each substrate, we measured the background fluorescence of soils and substrate and the quenching of MUB by soils, and used standard curves of MUB to calculate nmol of substrate hydrolyzed per hour per g of soil. Vegetation data were analyzed as relative percent cover for each species and also for functional groups (calculated as the sum of all component species).

For each ecosystem, we used variations on the metric H for determining homeostasis:

$$H\;=\frac{1}{m},$$

where *m* is the slope of log_e_ C:N_R_ or log_e_ C:P_R_ (resources) versus log_e_ C:N_B_ or log_e_ C:P_B_ (microbial biomass) (Cui et al., 2018). Strictly homeostatic organisms have an H of infinity, which presents analytical problems, and so the regression slope 1/H was used in its place (as in Persson et al., 2010). If the regression slope is not significant, the organisms are considered homeostatic (Persson et al., 2010).

CUE was estimated from the stoichiometry of the organic matter, microbial biomass, and extracellular enzyme activity (Sinsabaugh et al., 2016) for both C:N and C:P:

$${\text{CUE}}_{c:x}={\;\text{CUE}}_{\max}\left[\frac{S_{c:x}}{\left(S_{c:x}+{\;K}_{x}\right)}\right],$$

where the half‐saturation constant K_X_ was 0.5, and CUE_max,_ the upper limit for microbial growth efficiency based on thermodynamic constraints, was 0.6 (Sinsabaugh et al., 2016). S_C:X_ represents the offset by extracellular enzyme activity of the imbalance between the elemental composition of available resources and the composition of microbial biomass:

$$S_{c:x}=\left(\frac{1}{{\text{EEA}}_{c:x}}\right)\left(\frac{{\text{MB}}_{c:x}}{L_{c:x}}\right),$$

where L is the elemental composition of the substrate consumed (TOC, TN, or PO_4_). We used TOC, TN, and PO4 to represent substrates rather than total soil C, N, or P as water‐soluble nutrients likely represent a more sensitive measure of the soil substrate driving microbial activity (Mooshammer et al., 2014).

Our assays included potentials (EEA) or calculations based on potentials (CUE), in addition to comparison between different ecosystem processes. Therefore, we calculated the effect of long‐term nutrient addition on extracellular enzyme activity, CUE, and vegetation composition as the natural log of the response ratio (fertilized/control) for each block (Cusack, Torn, Mcdowell, & Silver, 2010). Significant effect sizes were determined based on their difference from zero (*α* = 0.05).

## 3 RESULTS

**Figure 1:**
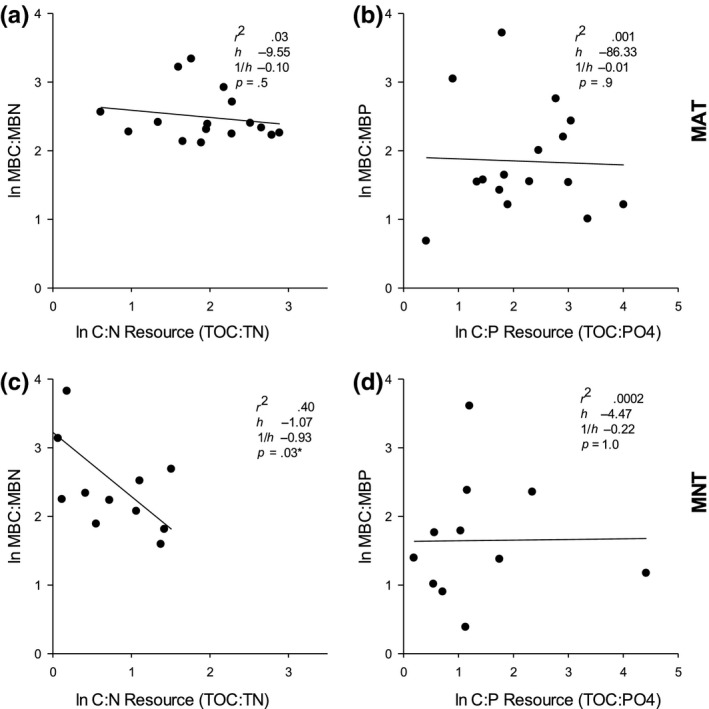
Scatter plots of log‐transformed soil available versus microbial biomass C:*N* (a, c) or C:P (b, d), where variation in soil resources is driven by long‐term N and P factorial fertilization treatments in moist acidic tundra (MAT: a,b) and moist nonacidic tundra (MNT: c,d) at the Arctic LTER at Toolik Lake, Alaska. Each point represents an individual plot in the fertilization experiment and the black line indicates slope of the regression regardless of significance—a significant relationship indicates nonhomeostasis in the microbial resource ratio (c only)

Microbial biomass C:N:P homeostasis was evident in the MAT ecosystem, for both C:N and C:P (Figure 1a,b) and in the MNT system, for C:P (Figure 1d). In all three of these results, homeostasis is assumed because the slopes of the organism: resource stoichiometries are not different from zero. In contrast, in the MNT, microbial biomass C:N declined with resource C:N, indicating no microbial homeostasis in this system: Microbes reduced their biomass N concentration in N‐rich soils (Figure 1c).

**Figure 2:**
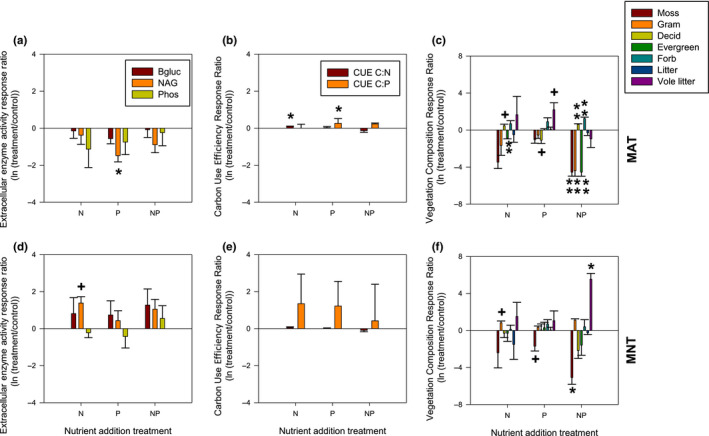
Mean (± SE) response ratio for extracellular enzyme activity (a, d), carbon use efficiency (b, e), and plant functional group abundance (c, f) for three nutrient addition treatments (N, P, and NP combined) relative to control plots in a long‐term fertilization experiment in moist acidic tundra (MAT: a‐c) and moist nonacidic tundra (MNT: d‐f) at the Arctic LTER at Toolik Lake, Alaska. A significant or trending response ratio (testing difference from 0 for each variable in each treatment) is indicated as + (*p* < .1), * (*p* < .05), and ** (*p* < .01)

Response ratios assess resource treatment relative to control, but also allow comparison between ecosystem‐level effects. Our combined plant and microbial analyses indicate that, in both ecosystems, plant community changes generated since the inception of the nutrient addition have a higher response ratio than microbial EEA or CUE (Figure 2a‐f).

In the MAT with the addition of N alone, microbial CUE‐N increased (Figure 2b), deciduous shrubs increased in relative abundance, and evergreens decreased (Figure 2c). With the addition of P alone, microbial NAG activity declined (Figure 2a), microbial CUE‐P increased (Figure 2b), there was a trend for deciduous shrubs to decline and vole litter increased (Figure 2c). With the addition of both N and P, all responses were in the plant community: deciduous shrubs and forbs increased, and mosses, graminoids and evergreens declined in relative abundance (Figure 2c).

In the MNT with the addition of N alone, there was a trend for NAG to increase (Figure 2d) and graminoid relative abundance to increase (Figure 2f). With P addition alone, there was no detected microbial homeostatic response and only a trend for moss abundance to decline (Figure 2f). With the addition of both N and P, all responses were again in the plant community: mosses declined and vole litter increased (Figure 2f).

## 4 DISCUSSION

The mechanistic portion of our hypothesis, that a strong plant response would reduce the variation in the stoichiometry of resources also reducing the microbial efforts to maintain homeostasis, was not supported. The ecosystem with the stronger plant community response (MAT) also had a wider range of soil resource stoichiometry (Figure 1), and there was no indication that microbial effort toward stoichiometry was lower in this community (Figure 2). Instead, the microbial efforts to maintain homeostasis, as measured by changes in CUE and potential enzyme activity, were generally low in both ecosystems (compared to the plant response) but significant in the ecosystem with the stronger plant response (MAT). In the MAT, there was an increase in CUE (C:N) with N additions and CUE (C:P) with P additions, supporting microbial shifts in use efficiencies with resource shifts (Mooshammer et al., 2014). There was also a decrease in NAG production (extracellular enzyme which supports microbial N acquisition) with P additions in the MAT, possibly in response to decreased N requirements for P uptake or enzyme synthesis.

We present our lack of support for the mechanistic hypothesis not as confirmation that these mechanisms are not important, but as a call to further research, in plant–soil systems with either less variance or larger replication. Inherent variability in the microbial C:N, or especially C:P ratios, temper our conclusions of homeostasis in both ecosystems, but a lack of ecosystem‐level research leaves us unable to conclude whether this variability is unusual. We are not aware of other studies examining mechanisms for microbial homeostasis that occur in environments where the both soil resource variability and plant composition vary strongly between areas on the same soil type. Studies in which both plant communities and soil resources vary in concert include either shifts in vegetation composition across strong environmental gradients (e.g., elevational gradients—Nottingham et al., 2015) or only subtle changes in vegetation communities with changing soil resources (e.g., shifts within a plant functional group—Griffiths, Spilles, & Bonkowski, 2012). Our two long‐term study systems have a replication level (*n* = 3 or 4) that is not unusually low compared with other similar long‐term studies, and this level of replication has been sufficient for numerous investigations with significant results over the past three decades (Chapin, Shaver, Giblin, Nadelhoffer, & Laundre, 1995; Hobbie et al., 2005; Koyama, Wallenstein, Simpson, & Moore, 2013; Mack, Schuur, Bret‐Harte, Shaver, & Chapin, 2004; McLaren & Buckeridge, 2019). Nonetheless, higher levels of replication may be necessary for studies that examine coupled responses of vegetation and soil communities. Where possible, higher levels of replication would also improve mechanistic insight into homeostasis, for instance to move beyond binary (yes/no) responses and instead allow researchers to assess the degree of variation in biomass stoichiometry, in different systems.

We found that the MAT ecosystem with a stronger plant biomass and community response (Figure 2; McLaren & Buckeridge, 2019), showed microbial homeostasis for both N and P whereas microbes in the MNT, with a weaker plant response, showed nonhomeostatic behavior for N and homeostasis only for P. However, high heterogeneity in fertilized natural systems may also make currently used metrics of homeostasis inappropriate. In the MAT, the homeostatic relationship for P was much weaker than that for N due to the very high variability in microbial C:P irrespective of resource C:P. In the MNT, we also saw very high variability in microbial C:P. According to frequently used metrics for homeostasis, these three relationships (MAT C:N and C:P and MNT C:P) are defined as homeostatic—variations in soil element ratios do not significantly affect microbial biomass element ratios because the slopes of the regressions (Figure 2) do not significantly differ from zero (Persson et al., 2010). However, this metric of homeostasis does not distinguish between strict homeostasis (changes in resource stoichiometry has no influence on organism stoichiometry (Sterner & Elser, 2002)) and those where the microbial stoichiometry is highly variable but also not dependent on resource stoichiometry. Persson et al. (2010) used a meta‐analysis approach to examine whether studies with nonsignificant slopes may have been misclassified as homeostatic, by using the residual variation in the datasets that had a significant regression fit (i.e., classified as nonhomeostatic, as with Figure 2b‐d) as an estimate of background variation, and then comparing this with the variation in the homeostatic datasets. With this approach, Persson et al. (2010) determined that for most of the species they examined, the homeostatic relationships were correctly classified. However, in studies with a more limited dataset such as ours, for which estimating background variation in this way is difficult, we propose an alternate index of variation (i.e., including a minimum *R* ^2^) should be used to define homeostasis. Although the large spread in the resource C:P in our study should be ideal for such a determination, many more data points are needed and are unavailable in this or most long‐term experimental manipulations. Therefore, we do not believe that our data provide sufficient evidence of homeostasis.

**Figure 3:**
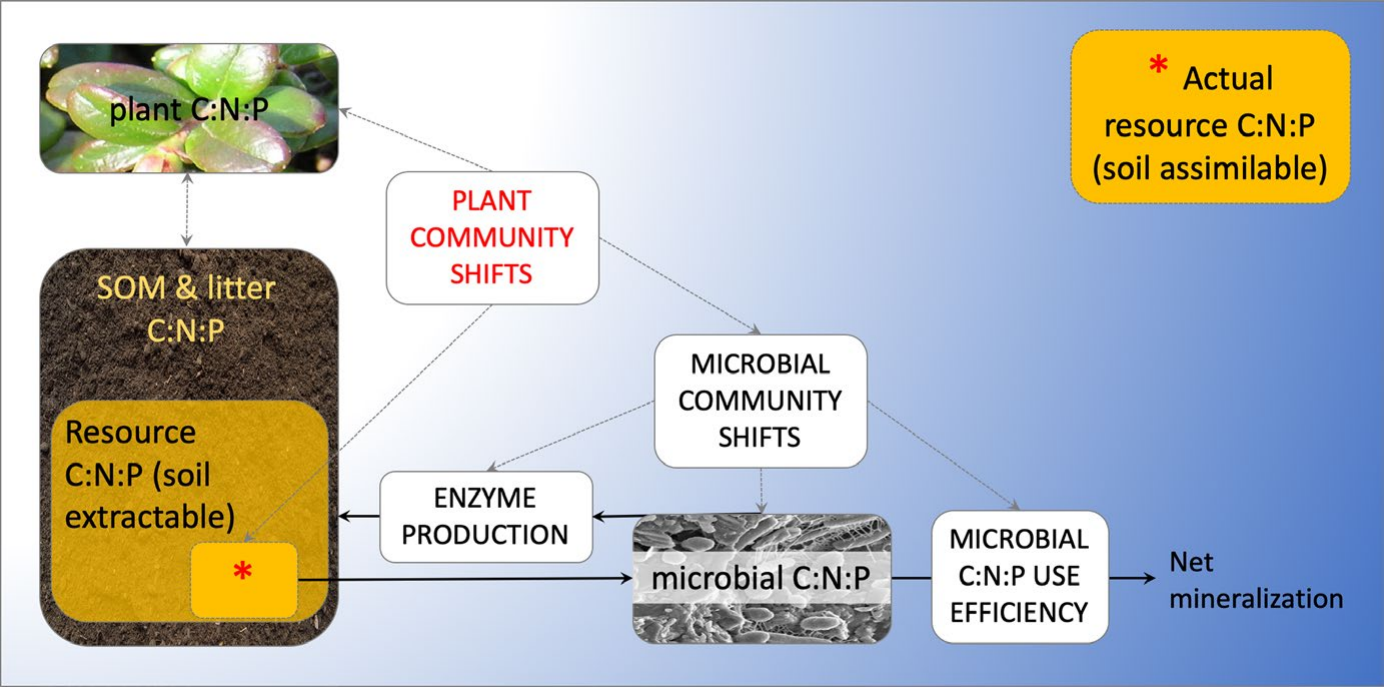
Schematic of mechanisms (white background) that control pool sizes and their C:N:P stoichiometry (color background) modified from Mooshammer et al. (2014) to include the relevance of plant community shifts for ecosystem‐level studies, and the effect of plant community shifts on the soil extractable versus microbial assimilable resource pool

Finally, we propose that concepts and methods with which ecologists currently define stoichiometry may not be relevant at the microbial scale. Specifically, soil resource C:N:P is an operational name, characterized by the total organic C and the inorganic and/or organic N and P that is extractable in the soil solution. The actual pool of C (and N and P if organic N and P were included) that is used in this calculation varies by extraction protocol and soil type. These pools (especially C) undoubtedly contain large and variable amounts of C, N, or P that are not directly assimilable by soil microbes (Figure 3). Plants may alter this assimilable pool C:N:P, both indirectly and directly, by the mechanisms outlined in the paragraph in the introduction of this study, including nutrient inputs, outputs, and shifting plant–microbial associations. For example, in a study across 9 different soil type and vegetation community combinations, microbes maintained homeostasis partially through changing EEA stoichiometry, which was regulated more strongly by the characteristics of the plant community than soil physiochemical variables (Cui et al., 2018). Shifts in plant communities within the same soil type such as we investigate in this study may produce similar soil resource C:N:P between control and resource‐amended communities, but may have very different assimilable pool C:N:P, and thus may result in different microbial mechanisms used to maintain homeostasis. We encourage greater understanding of the C available to microbes from the soil resource pool. In much the same way that we measure inorganic N and P, we can dig deeper into the microbial resource C pool at a molecular scale. A number of methodological improvements already exist in the literature, including (1) isotopic tracer methods of low molecular weight (i.e., assimilable) carbon (Lynch, Machmuller, Cotrufo, Paul, & Wallenstein, 2018); (2) size‐based filtration fractionation of soil extractions (Farrell, Hill, Farrar, Bardgett, & Jones, 2011); (3) molecular‐level exploration of C quality in soil extractions (i.e., with HPLC, GC/MS, nanoSIMS, NMR; Hall et al., 2011); or (4) a companion incubation of the soil extract to assess the bioavailable fraction. Any of these methods could then be used to scale the C, N, or P content in the C:N:P ratio. This consideration and those suggested above should improve process‐level assessment of microbial response to soil resource C:N:P across scales.

In summary, we proposed that ecosystems with strong plant community response to fertilization would allow maintenance of microbial homeostasis with minimal microbial effort. We found little support for our hypothesis because although we found higher indications of microbial homeostasis in the ecosystem with stronger plant community response to steep resource gradients, these did not appear to be dictated by less microbial resource cost. Therefore, we remain uncertain to what extent plant community dynamics impact microbial homeostasis in ecosystems with changing plant communities across strong resource gradients. Our results highlighted issues with variability in ecosystem‐level experimental systems of microbial homeostasis with a strong plant community response on the same soil type, and potential issues with how we quantify the microbial assimilable pool of soil resources. We respond with a call for further ecosystem‐level investigations of microbial homeostasis where resource gradients exist on the same soil type in natural ecosystems, such as those in long‐term nutrient addition experiments. We suggest using designs that increase field‐level replication, isolate potential plant–microbial associations, and enhance the molecular‐level quantification of the microbial assimilable resource pool.

## AUTHOR CONTRIBUTIONS

**Kate M. Buckeridge:** Conceptualization (lead); Soil analyses (perform); Statistical analyses (perform); Writing–original draft (lead). **Jennie R. McLaren:**Conceptualization (lead); Fieldwork (perform); Statistical analyses (perform); Writing–original draft (lead).

## Supporting information

Table S1

## Data Availability

Vegetation and soil data are available publicly at: Species cover: Arctic Data Center Entry http://dx.doi.org/10.6073/pasta/8a2999c9ed297a184aaca7057e1ae177. Soil microbial biomass C, N and P; extracellular enzyme activity, soil extractable C, N and P; soil total C, N and P, in g/m^2^ (Appendix Table S1) and µg/g: Arctic Data Center Entry https://doi.org/10.6073/pasta/2302b3a5eab56970aa4e4f71d36b7fce.
