# Supplementary material for: Does plant community plasticity mediate microbial homeostasis?
Source: Ecol Evol. 2020 Apr 24;10(12):5251–8. doi: 10.1002/ece3.6269 (PMC7319231; doi:10.1002/ece3.6269)
Supplement: Supplementary file 1 — Table S1 [file ECE3-10-5251-s001.pdf]

Means and standard error (in italics, below means) for the soil total, microbial biomass and soil water-extractable carbon (C), nitrogen (N) and phosphorus (P), and the extracellular enzyme activity for beta-glucosidase (BG), N-acetyl-glucosaminidase (NAG) and phosphatase (Phos), for the two ecosystems in this study, the moist acidic tundra (n=4) and moist non-acidic tundra (n=3). ‘Addition’ represents the long-term nutrient additions of nitrogen (N: 10 g m^{-2} y^{-1}), phosphorus (P: 5 g m^{-2} y^{-1}), or combined nitrogen and phosphorus (N + P). These values represent the depth of the organic horizon, which is deeper in the MNT than the MAT. Further soil characteristics can be found in the online data repository.

		Total (g m^{-2})			Microbial Biomass (g m^{-2})			Extractable (g m^{-2})			Extracellular enzyme activity (mmol m^{-2} h^{-1})		
Ecosystem	Addition	C	N	P	C	N	P	C	N	P	BG	NAG	Phos
Moist Acidic Tundra (MAT)	Control	5689	173	12.7	9.07	0.75	0.66	27.5	2.59	0.43	0.75	1.89	2.19
		1083	*36.0*	*3.00*	*1.13*	*0.18*	*0.22*	*3.7*	*0.61*	*0.08*	*0.22*	*1.08*	*0.56*
	N	5438	229	13.0	7.23	0.43	0.52	18.8	4.70	0.50	0.64	0.60	1.54
		1212	*66.0*	*4.65*	*1.73*	*0.04*	*0.15*	*7.2*	*1.11*	*0.16*	*0.19*	*0.26*	*0.46*
	P	6215	179	30.3	9.63	0.75	0.91	28.2	1.90	3.97	0.43	0.68	0.62
		786	*21.4*	*9.92*	*2.94*	*0.23*	*0.37*	*3.3*	*0.47*	*1.06*	*0.11*	*0.20*	*0.28*
	N + P	5735	274	26.5	7.68	0.57	0.46	31.4	4.41	1.95	0.66	1.11	0.85
		594	*27.6*	*5.46*	*0.84*	*0.15*	*0.14*	*5.0*	*0.96*	*0.27*	*0.14*	*0.27*	*0.16*
Moist Non-acidic Tundra (MNT)	Control	22093	1277	50.7	20.9	1.45	1.87	32.3	3.08	1.56	4.28	7.70	1.49
		15311	*974*	*31.8*	*11.0*	*0.51*	*1.62*	*9.3*	*0.59*	*0.68*	*3.13*	*4.70*	*0.95*
	N	12253	547	27.6	13.4	0.21	0.41	27.2	10.3	2.39	4.86	4.76	4.49
		815	*34.7*	*2.55*	*6.84*	*0.04*	*0.16*	*9.1*	*4.15*	*1.44*	*0.69*	*1.74*	*1.53*
	P	13644	567	43.4	15.9	1.68	2.10	23.7	4.32	3.81	4.47	3.56	1.82
		3437	*113*	*15.5*	*5.24*	*0.43*	*0.75*	*8.5*	*2.05*	*1.62*	*0.57*	*0.83*	*0.59*
	N + P	19657	1101	90.7	20.9	2.42	2.51	25.0	6.22	6.27	7.55	8.87	3.08
		4373	*212*	*18.6*	*9.67*	*1.39*	*1.57*	*4.6*	*1.29*	*1.02*	*0.52*	*0.74*	*0.74*
